# Energy Consumption of Air-Separation Adsorption Methods

**DOI:** 10.3390/e20040232

**Published:** 2018-03-28

**Authors:** Tomasz Banaszkiewicz, Maciej Chorowski

**Affiliations:** Department of Cryogenic, Aeronautic and Process Engineering, Wroclaw University of Science and Technology, 50-370 Wroclaw, Poland

**Keywords:** oxygen, adsorption, separation, energy engineering

## Abstract

Adsorption technology is currently one of the most popular methods of air separation. At relatively low energy expenditure, this allows oxygen to be obtained with sufficient purity for oxyfuel, metallurgy or medical applications. The adsorption process is dependent on several factors such as pressure, temperature, the concentration of adsorbed element in the gas phase, or the surface area of the phase boundary. The paper shows the calculation of the minimum energy needed for oxygen separation taking into account the advantages and disadvantages of the adsorption methods. The article shows how many times the energy consumption of a real oxygen-separation plant is higher than the theoretical energy consumption, and indicates which components of the adsoption installation can be further improved. The paper is supported by research conducted on an oxygen-separation installation at a semi-technical scale.

## 1. Introduction

Oxygen is a crucial gas used in many industrial processes such as metallurgy, biotechnology, medicine and emerging carbon capture and storage (CCS) technologies. At present, on an industrial scale, oxygen is obtained in particular through the processes of condensation and distillation of atmospheric air which occur in cryogenic air-separation units. The cryogenic technology makes it possible to obtain oxygen of over 99% purity. An additional advantage of the cryogenic technology is obtaining a high purity of the remaining ingredients of air, especially nitrogen and argon, and in some cases also krypton and xenon. To obtain lower purity oxygen (of about 95%), membrane and adsorption techniques are used [1,2]. However membrane technologies are still in pilot stages with capacities not exceeding several tens of tons per day [3,4]. Hence, there are two mature technologies for separating oxygen from air whose efficiency and purity of separated oxygen are sufficient for the needs of industry—cryogenic and adsorptive.

Increasingly, a demand for oxygen occurs in technologies showing a relatively low oxygen demand (up to 1000 tons per 24 h), such as biotechnological processes. With such performances, the operating costs of the cryogenic system make it unprofitable. For such oxygen capacities, technologies based on the adsorption process can be competitive to cryogenic technologies. Adsorption methods allow the use of various energy sources in the process of oxygen separation [5,6]. The pressure swing adsorption (PSA) process, which is best described in literature, is based on gas compressors requiring electrical energy. Another form of energy is applied by the temperature swing adsorption (TSA) process which requires a supply of heat for the stages of adsorption-bed regeneration.

The minimum work of gas-mixture separation is the sum of works of isothermal reversible compression of all (*k*) components from their partial pressures (*p_i_*) to the pressure of the mixture (*P*). On the assumption that gas properties may be described by means of an equation of the ideal gas condition, and *y_i_* is the mole fraction of the *i*-th component in the total mixture, we obtain the following formula:

(1) $$W=\;\sum_{i=1}^{k}\int_{Pi}^{P}-PdV=\;\sum_{i=1}^{k}-\int_{Py_{i}}^{P}PdV=\;\sum_{i=1}^{k}n_{i}RT\ln\left(\frac{1}{y_{i}}\right),$$

The minimum work of separating oxygen from the air in standard conditions (calculated using Equation (1)) is approximately 192 MJ (51.3 kWh) per ton of pure oxygen.

## 2. Energy Consumption of Oxygen Separation from the Air

The minimum energy consumption calculated above refers to the ideal theoretical process. It does not include a real way of conducting the separation of the gas mixture. Similar calculations of the energy consumption minimum can be carried out for each of the separation methods by taking into account its advantages and limitations.

In the adsorption technology, the separation of oxygen from air is most often carried out with the use of zeolite beds. Research on the technology of adsorptive air separation presented here was conducted with the use of 5A zeolite bed.

To describe isotherms of oxygen and nitrogen adsorption from air, the DSL model (dual-site Langmuir) was applied [7,8]:

(2) $$a_{O_{2}}=a_{md}\frac{K_{dO_{2}}p_{O_{2}}}{1+K_{dO_{2}}p_{O_{2}}+K_{d\;N_{2}}p_{N_{2}}}+a_{mb}\frac{K_{bO_{2}}p_{O_{2}}}{1+K_{bO_{2}}p_{O_{2}}+K_{bN_{2}}p_{N_{2}}}$$

(3) $$a_{N_{2}}=a_{md}\frac{K_{d\;N_{2}}p_{N_{2}}}{1+K_{dO_{2}}p_{O_{2}}+K_{dN_{2}}p_{N_{2}}}+a_{mb}\frac{K_{b\;N_{2}}p_{N_{2}}}{1+K_{bO_{2}}p_{O_{2}}+K_{bN_{2}}p_{N_{2}}}$$

where *a_mb_*, and *K_bi_* are the saturation capacity and the affinity parameter on the first set of sites, respectively, and *a_md_*, and *K_di_* are the analogous parameters on the second set of sites. By convention, we assume that the first set of sites has the larger affinity parameter, i.e., $K_{bO_{2}}$ > $K_{dO_{2}}$.

In order to complete the description of the DSL isotherm, it is considered that the temperature dependence of *K_d_* and *K_b_* constants is performed according to the Arrhenius equation [9]:

(4) $$K_{bi}=K_{bi}^{0}e^{\frac{Q_{bi}}{R}\left(\frac{1}{T}\;-\;\frac{1}{T_{0}}\right)}\;K_{di}=K_{di}^{0}e^{\frac{Q_{di}}{R}\left(\frac{1}{T}\;-\;\frac{1}{T_{0}}\right)}$$

where $K_{bi}^{0}$ and $K_{di}^{0}$ are the two affinity parameters of *i*-th gas at the reference temperature of *T*_0,_ and *Q_bi_* and *Q_di_* is the heat of adsorption of *i*-th gas on the two sites.

To simplify the calculations, it is possible to use Dalton’s law which says that the pressure (*P*) exerted by the gas mixture is the sum of the partial pressures (*p_i_*):

(5) $$P=\overset{k}{\underset{i=1}{\sum }}p_{i}$$

In further simplifications, it can be assumed that the partial pressure of each component of air is equal to the total pressure of air multiplied by the mole fraction (*y*) of the particular component. Thus, the following equations can be used for the calculation methods described above:

(6) $$p_{O_{2}}=Py_{O_{2}}\;\;p_{N_{2}}=Py_{N_{2}}\;$$

After applying Equations (4)–(6), the dual-site Langmuir model of isotherms takes the following form:

(7) $$a_{O_{2}}=a_{md}\frac{K_{dO_{2}}Py_{O_{2}}}{1+K_{dO_{2}}Py_{O_{2}}+K_{d\;N_{2}}Py_{N_{2}}}+a_{mb}\frac{K_{bO_{2}}Py_{O_{2}}}{1+K_{bO_{2}}Py_{O_{2}}+K_{bN_{2}}Py_{N_{2}}}$$

(8) $$a_{N_{2}}=a_{md}\frac{K_{dN_{2}}Py_{N_{2}}}{1+K_{dO_{2}}Py_{O_{2}}+K_{dN_{2}}Py_{N_{2}}}+a_{mb}\frac{K_{bN_{2}}Py_{N_{2}}}{1+K_{bO_{2}}Py_{O_{2}}+K_{bN_{2}}Py_{N_{2}}}$$

In order to determine the parameters of Equations (7) and (8) the measuring points of adsorption isotherms of pure nitrogen and oxygen on zeolite 5A were used. The isotherms of the pure gases are presented in Table 1 and Table 2.

The first step in determining the parameters of the DSL model was to make affinity parameters (which in this forms are simply the equilibrium constants) independent of the temperature and composition:

(9) $$a_{O_{2}}=a_{md}\frac{K_{dO_{2}}^{0}P}{1+K_{dO_{2}}^{0}P}+a_{mb}\frac{K_{bO_{2}}^{0}P}{1+K_{bO_{2}}^{0}P}$$

(10) $$a_{N_{2}}=a_{md}\frac{K_{dN_{2}}^{0}P}{1+K_{dN_{2}}^{0}P}+a_{mb}\frac{K_{bN_{2}}^{0}P}{1+K_{bN_{2}}^{0}P}$$

On the basis of the data from Table 1 and Table 2, the values of the parameters of Equations (9) and (10) were determined. The equilibrium constants of K^0^ were determined separately for the adsorption temperature equaling 23 °C and the adsorption temperature equaling 45 °C. Next, the values of the adsorption heat for the particular areas of the DSL model were determined with the use of Equation (4).

Below in Table 3 the values of the DSL model parameters are presented.

**Figure 1:**
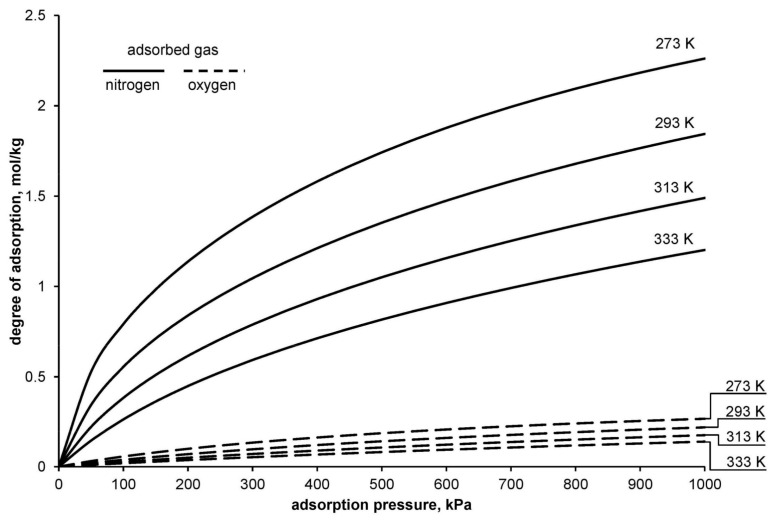
Nitrogen and oxygen adsorption isotherm on zeolite 5A (Dual-site Langmuir (DSL) model).

Figure 1 presents a chart of exemplary nitrogen and oxygen adsorption isotherms determined with the use of the DSL model.

As shown on Figure 1, while passing a portion of air through the zeolite bed, both nitrogen and a small amount of oxygen are subject to the process of adsorption. Adsorption of oxygen on the adsorption bed forces an increased use of energy, both in the compression process (adsorption process) and in the process of bed regeneration. This effect should be included in the calculations of energy consumption of the adsorptive technology of oxygen separation from air.

**Figure 2:**
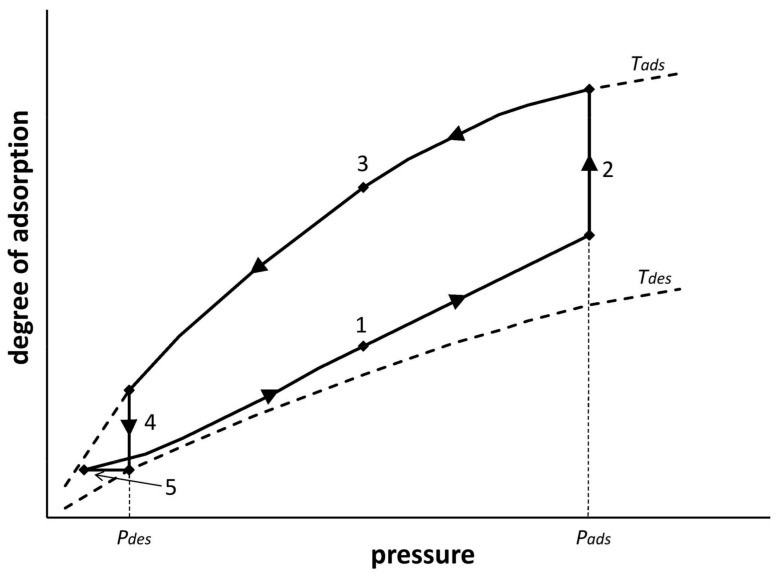
Adsorption cycle of the Vacuum–Pressure–Temperature Swing Adsorption (VPTSA) process in single adsorption bed (*P_ads_*—adsorption press; *P_des_*—desorption press; *T_ads_*—adsorption temp.; *T_des_*—desorption temp.; 1—pressurization; 2—oxygen separation; 3—depressurization; 4—adsorption bed heating; 5—adsorption bed cooling).

Calculations of the minimum energy consumption of oxygen separation on the 5A zeolite bed were made on the basis of determined isotherms of nitrogen and oxygen adsorption from air—Equations (8) and (9). The calculations were made for adsorption in Vacuum–Pressure–Temperature Swing Adsorption (VPTSA) technology as the most general. Figure 2 shows the adsorption cycle of the VPTSA process carried out in single adsorption bed. The shape of the VPTSA cycle line shown in Figure 2 was obtained in the experimental research process on the apparatus described in Chapter 3.

In order to determine the minimal energy consumption of oxygen separation on the adsorption bed, the following calculations were made.

First, the number of nitrogen moles that can be adsorbed in the mass unit of an adsorbent in a single adsorption cycle was calculated. Calculations were made for different adsorption pressures and different temperatures of the bed regeneration. In the calculations, we used adsorption isotherms by subtracting a degree of nitrogen adsorption after the adsorption stage from a degree of nitrogen adsorption after the desorption stage:

(11) $$n_{N_{2}}=a_{adsN_{2}}-a_{desN_{2}}$$

In a similar way, the amount of adsorbed oxygen on the bed during the same adsorption cycle was determined:

(12) $$n_{O_{2}}=a_{adsO_{2}}-a_{desO_{2}}$$

The next step was to determine the minimal portion of air which must be pumped into the adsorption tank in order to obtain the previously determined amount of adsorbed nitrogen. This portion of air was calculated on the assumption that all nitrogen from the air would be adsorbed on the adsorption bed.

(13) $$n_{air}=\frac{n_{N_{2}}}{0.78}$$

Next, the amount of separated oxygen with a purity of 95% (the remaining 5% is mainly argon) was determined in a balance as a difference between the number of air molecules flowing into the adsorber and the number of all adsorbed molecules.

(14) $$n_{95\%O_{2}}=n_{air}-\left(n_{N_{2}}+n_{O_{2}}\right)$$

In the VPTSA process, energy should be delivered in two stages. The adsorption stage is carried out at an increased pressure. During each adsorption stage, air portion compression from the ambient pressure to the adsorption pressure takes place. Regeneration of the adsorptive bed in the VPTSA process is carried out by lowering the pressure to the pressure of desorption, and heating the adsorption bed from the ambient temperature up to the particular temperature of the bed regeneration. It is therefore necessary to deliver work to the adsorption stage, and heat to the desorption stage (adsorption-bed regeneration). During the adsorption stage, work is delivered by compressing the air from desorption pressure (*P_des_*) to adsorption pressure (*P_ads_*). During the regeneration process, the heat is transferred directly into the adsorption.

To determine the work of compression, the following equation was used:

(15) $$W_{C}=n_{air}RT_{ads}\ln\left(\frac{P_{ads}}{P_{des}}\right)$$

The compression work was converted into work per mass unit of separated oxygen with a purity of 95% (other 5% is argon):

(16) $$W_{C}^{*}=\frac{W_{C}}{n_{O_{2}}\left(0.95M_{O_{2}}+0.05M_{Ar}\right)}$$

It is worth noting that the portion of air (*n_air_*) that is being compressed in the adsorption stage depends on the conditions at the end of the regeneration stage as well as on the conditions at the adsoption stage. This means that the work of compression depends both on the pressure and temperature of the adsorption stage, but also on the pressure and temperature of the bed-regeneration stage.

**Figure 3:**
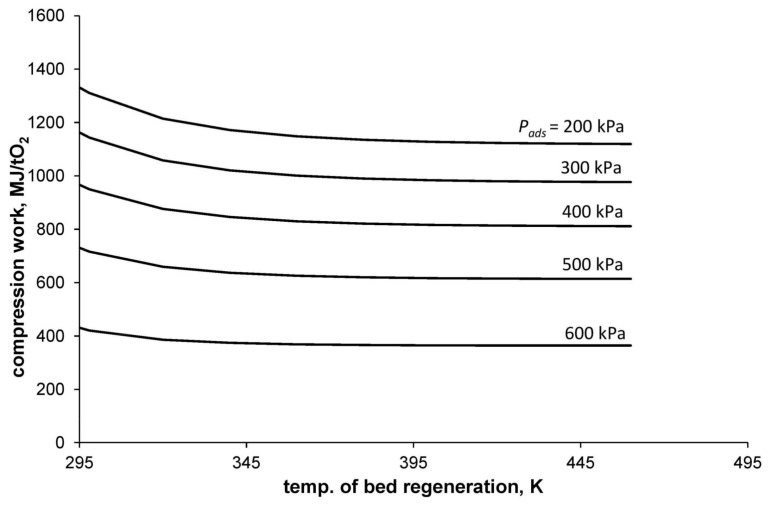
Minimum work of air compression.

Figure 3 shows the dependence of the compression work per mass unit of separated oxygen with a purity of 95% ($W_{C}^{*}$) on the bed-regeneration temperature and the adsorption pressure. The calculations were made with the assumption that the adsorption temperature is equal to 295 K and the desorption pressure is equal to atmospheric pressure.

The heat required to regenerate the bed was calculated by assuming the average specific heat of zeolite *Cp_zeol_* = 800 J/kgK [11], the specific heat of air *Cp_pow_* = 1000 J/kgK, and knowing the temperature of the bed regeneration. The heat of adsorption can be neglected in the calculation since it is several times lower in than the heat supplied to the adsorption bed in the regeneration stage. In order to calculate the heat required for the regeneration stage, the following equations were used:

(17) $$W_{h}=\left(m_{zeol}Cp_{zeol}+n_{air}M_{air}Cp_{air}\right)\Delta T$$

Equation (17) was converted by using the number of moles of oxygen ($n_{O_{2}}$) which was obtained from one kilogram of adsorbent. Thus, the heat of the regeneration bed charged per mass unit of the produced oxygen of a required purity (95%) was obtained:

(18) $$W_{h}^{*}=\frac{\left(Cp_{zeol}+n_{air}M_{air}Cp_{air}\right)\Delta T}{n_{O_{2}}\left(0.95M_{O_{2}}+0.05M_{Ar}\right)}$$

**Figure 4:**
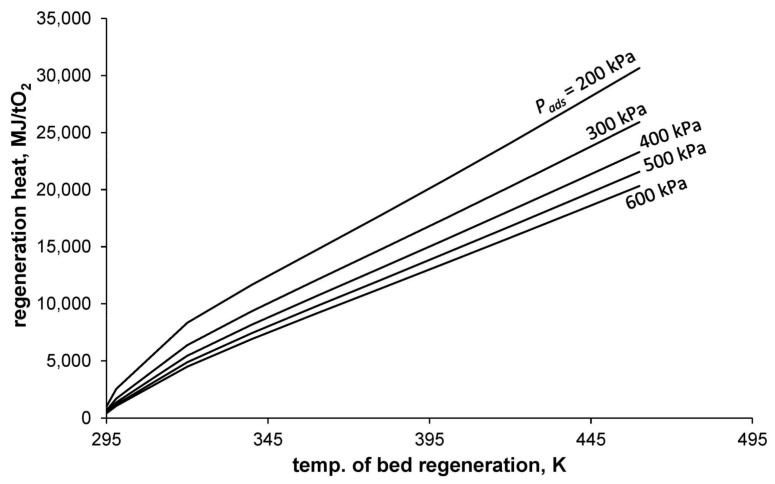
Heat consumption of oxygen separation for different temperatures of bed regeneration and different pressures of adsorption.

The dependence of regeneration heat (calculated using Equation (18)) on the process conducting conditions is presented in Figure 4.

The calculated energy consumption of oxygen separation from air with the use of the adsorption bed in the form of zeolite 5A is the minimal energy consumption required to obtain oxygen using this technology. Compared to the thermodynamic minimum of oxygen separation of 184.7 MJ/t O_2_, energy consumption calculated by taking into account the applied adsorption technology is at least two and a half times higher. Energy consumption of the oxygen-separation process by means of adsorption methods is strongly influenced by the process conducting conditions (adsorption pressure, desorption pressure, adsorption temperature, and bed-desorption temperature) as well as the type of a sorbent that is used.

Energy consumption determined from the thermodynamic minimum, which does not take into account the technology used, constitutes a global minimum of energy consumption of oxygen separation. For this reason, it is the basis for a comparative assessment of the energy consumption of different oxygen separation technologies.

## 3. Oxygen-Separation Energy Consumption Measurements

Studies on the energy consumption of oxygen separation from the air were made on a semi-technical scale. The apparatus was built as part of a project co-financed by the National Center for Research and Development. The apparatus was built as a tank installation working in Vacuum–Pressure Swing Adsorption (VPSA) technology with a capacity of 100 m^3^ of oxygen per hour (in normal conditions). This is sufficient performance for hospitals, sewage treatment plants and small smelters. The big advantage of the installation is that it is built in a standard-size container allowing full mobility of the fully assembled equipment, which is ready to work immediately after unloading.

**Figure 5:**
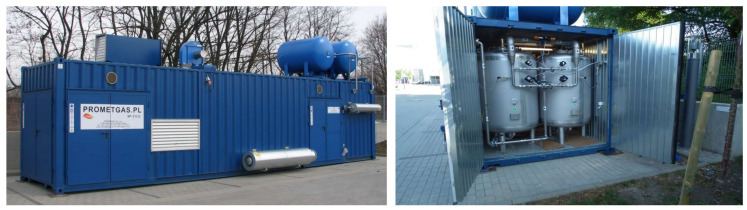
Mobile pilot installation Vacuum–Pressure Swing Adsorption (VPSA-O_2_) for extracting oxygen from the air.

The installation makes it possible to obtain oxygen with a purity of 95%. It uses zeolite molecular sieves characterized by a high selectivity for the adsorption of nitrogen in relation to oxygen. The installation is shown in Figure 5.

The air, which is taken from the environment through an air inlet sampling probe with a filter, is compressed in a Root’s blower to a pressure of 150 ÷ 180 kPa abs. The air is then cooled in a cooler supplied with water from the circulation of the chiller aggregate to a temperature of 25 ÷ 30 °C. A water condensate separated from the air is separated in the separator. The air flows to buffer tanks where it is temporarily stored.

The air prepared in this way is separated into oxygen having a purity of up to 95% in adsorbers working in the VPSA technology.

The VPSA process in this apparatus is carried out between an adsorption pressure of 150 kPa abs., and a desorption pressure of about 50 kPa abs.

**Figure 6:**
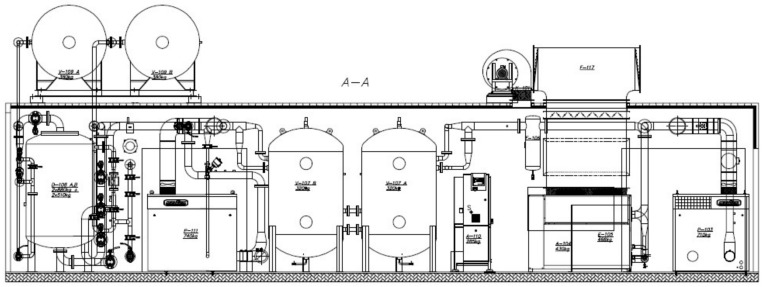
Disposition of devices in the container.

The disposition of devices in the container is shown in Figure 6.

**Figure 7:**
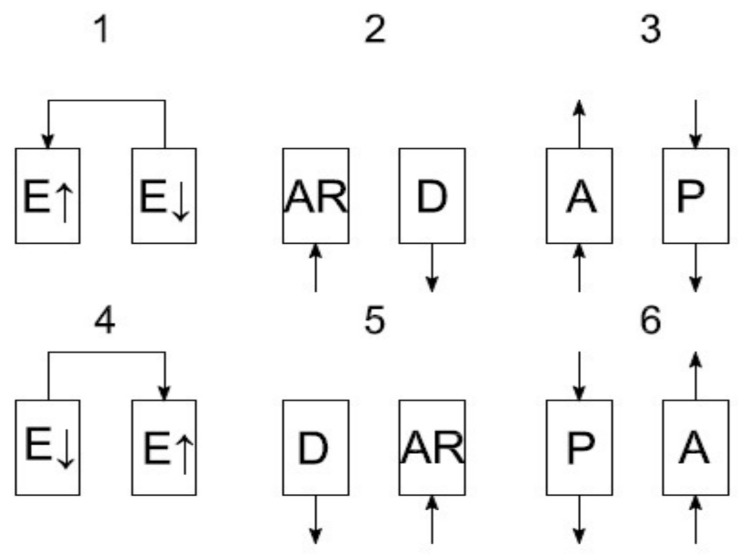
A scheme of the VPSA cycle carried out in the container installation of oxygen separation from air. Symbols used in the Figure 7: E↑/E↓—increasing, decreasing, equalizing pressures; AR—filling with air; A—adsorption; D—desorption; P—rinsing.

The process of adsorptive oxygen separation is conducted according to various recipes. The main idea of the device operation is based on the VPSA cycle. A general scheme of the recipe used in this apparatus is presented in Figure 7.

In the first phase, the pressure equalization takes place. Subsequently, the adsorber is filled with air until the adsorption pressure is obtained. After obtaining the adsorption pressure in the adsorber, the phase of oxygen separation takes place. In the phase of oxygen separation (adsorption) to the absorber, the air is supplied from which nitrogen is adsorbed and oxygen is delivered into the tank. At the same time, in the second adsorber there is a process of rinsing with oxygen from the adsorption line. Adsorption is carried out at a pressure of 150 kPa abs. Rinsing is carried out at a pressure of 40–50 kPa abs. This is the Skarstrom cycle carried out in 6 sequences, where sequences 4, 5, 6 are the mirror image of sequences 1, 2, 3.

**Figure 8:**
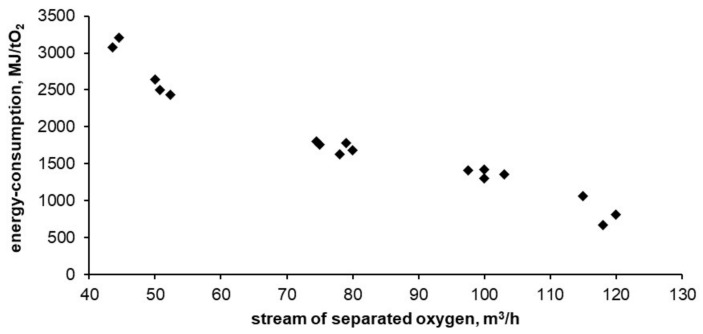
Dependence of energy consumption of separated oxygen on the apparatus performance.

**Figure 9:**
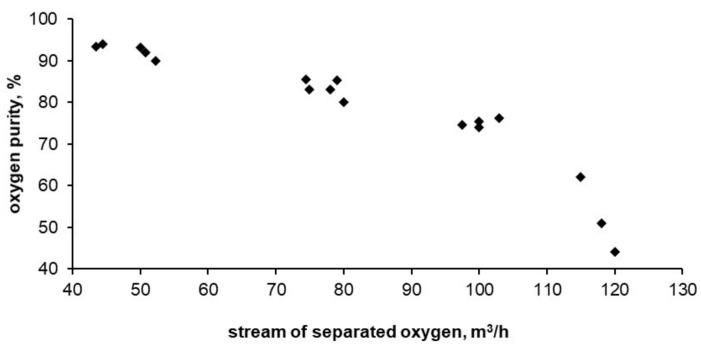
Dependence of separated oxygen purity on the apparatus performance.

**Figure 10:**
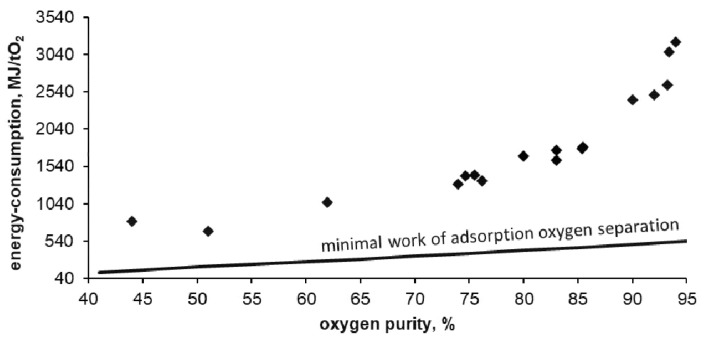
Dependence of energy consumption on separated oxygen purity.

Below, Figure 8, Figure 9 and Figure 10 show the results of tests carried out on the described adsorptive oxygen separator. The data show the relationship between energy consumption, oxygen purity and the stream of separated oxygen. Figure 10 shows the dependence of energy consumption of the apparatus on the purity of the obtained oxygen.

In the tested installation, the energy consumption of oxygen separation was 3200 MJ per ton of oxygen witch a purity of 94%. This is 6 times higher than the thermodynamic minimum that can be obtained for this technology (calculated using the DSL model). The lowest obtained energy consumption reached 670 MJ per ton of oxygen with purity of 50%. This is 3.5 times higher than the thermodynamic minimum that can be obtained for this technology. As expected, with the decrease in purity of the separated oxygen its unit cost is reduced. With reduced oxygen purity, the required energy is asymptotically approaching the thermodynamic minimum possible for adsorption technology.

All of the energy consumption, oxygen purity and efficiency values of the apparatus are summarized in Table 4.

## 4. Summary

The use of adsorption in air-separation technology enables us to obtain oxygen with a purity of up to 95%, where the main impurity is argon. Energy consumption of adsorption installations for air separation ranges from about 11,000 MJ/t O_2_ for laboratory units with small efficiencies up to about 1450 MJ/t O_2_ for large, optimized systems. In the tested apparatus, the energy consumption of oxygen separation reached 3200 MJ per ton of oxygen with a purity of 94%.

It is estimated, that energy consumption at about 1450–1800 MJ/ton of oxygen at oxygen purity equalling 95% can be achieved by reducing the energy consumption of peripheral devices (such as a dehumidifier, which in large adsorption systems can be regenerated by using heat recovered from the cooling compressors). Energy consumption at this level is comparable to the energy consumption of the cryogenic installation with a similar performance. An additional advantage of the adsorption installation is its mobility. The apparatus presented here is placed permanently in a standard-size container. This allows the separation and utilization of oxygen directly at the place where there is a demand for it.

Due to the cyclical nature of the processes and the lack of steady states in systems, energy consumption is strongly dependent on the recipes used and sequences of processes. Adsorption systems can be fully automated and unattended. Due to their relatively small sizes, adsorption systems can be designed as fully mobile units. Thanks to this, they can be used directly at a place of a temporary or test demand for oxygen. A great advantage of the adsorption systems is their short start-up time which, depending on the installation, varies from several minutes to several hours. Installations of adsorptive separation of oxygen from atmospheric air are characterized by relatively low investment costs which are competitive in relation to cryogenic installations with performances of 200–300 tons of oxygen per day. For higher performances, adsorption installations are not competitive.

## Figures and Tables

**Table 1:** Adsorption isotherm of pure nitrogen on zeolite 5A—experimental data [10].

Temp. = 23 °C		Temp. = 45 °C	
Pressure, kPa	Degree of Adsorption, mol/kg	Pressure, kPa	Degree of Adsorption, mol/kg
8.6	0.0894	12.0	0.0669
26.1	0.2272	36.8	0.1816
54.2	0.3987	73.0	0.3193
121.2	0.6951	153.9	0.5584
233.5	1.0345	278.0	0.8346
388.2	1.3574	438.3	1.1013
640.1	1.7085	693.6	1.4117
967.0	2.0129	1020.3	1.6951
1341.5	2.2529	1393.6	1.9298
1741.0	2.4371	1790.9	2.1158

**Table 2:** Adsorption isotherm of pure oxygen on zeolite 5A—experimental data [10].

Temp. = 23 °C		Temp. = 45 °C	
Pressure, kPa	Degree of Adsorption, mol/kg	Pressure, kPa	Degree of Adsorption, mol/kg
20.3	0.0407	22.1	0.0281
47.6	0.0941	60.3	0.0767
96.3	0.1806	107.1	0.1344
194.5	0.3557	205.5	0.2484
329.3	0.5603	339.8	0.3954
534.0	0.8336	499.9	0.5553
786.3	1.1154	749.7	0.7773
1080.1	1.3861	1062.0	1.0157
1390.2	1.6213	1416.0	1.2443
1766.1	1.8436	1796.8	1.4499

**Table 3:** Dual-site Langmuir (DSL) isotherm coefficients.

Parameter	Nitrogen	Oxygen
*a_mb_*, mole/kg	0.7	
$K_{b}^{0}$, bar^−1^ *	1.214	0.051
*Q_b_*, J/mole	22,864.53	15,561.08
*a_md_*, mole/kg	3.2	
$K_{d}^{0}$, bar^−1^ *	0.073	0.051
*Q_d_*, J/mole	17,859.21	15,561.28

* Equilibrium constants determined for temperature *T*_0_ = 23 °C.

**Table 4:** Physical quantities measured during tests of the container installation for air separation.

Oxygen Stream, m^3^ O_2_/h	Energy Consumption, MJ/t O_2_	Oxygen Purity, %
43.5	3070	93.4
44.5	3211	94.0
50.0	2635	93.2
50.8	2498	92.0
52.3	2437	90.0
74.5	1796	85.5
75.0	1756	83.0
78.0	1627	83.0
79.0	1774	85.4
80.0	1684	80.0
97.5	1411	74.7
100.0	1303	74.0
100.0	1418	75.5
103.0	1350	76.2
115.0	1062	62.0
118.0	669	51.0
120.0	806	44.0

## Nomenclature

*a*	adsorption capacity, mole/kg
*a_m_*	saturation capacity, mole/kg
*Cp*	specific heat, J/kgK
*K*	affinity parameter, bar^−1^
*K*^0^	affinity parameter known for given temperature *T*_0_, bar^−1^
*m*	mass, kg
*M*	molar mass, kg/kmole
*n*	moles, mole
*p*	partial pressure, Pa
*P*	pressure of mixture, Pa
*Q*	heat of adsorption, J/mole
*R*	universal gas constant, J/mole K
*T*	temperature, K
*V*	volume, m^3^
*W*	work, J
*W**	work given per mass of the separated oxygen, J/kg
*y*	mole fraction

## Subscripts

95%	purity of 95%
*ads*	adsorption
*air*	air mixture
*Ar*	pure argon
*b*, *d*	respectively, first and second set of sites in DSL model
*c*	compression
*des*	desorption
*h*	heat
*i*	*i*-th element of the mixture
N_2_	pure nitrogen
O_2_	pure oxygen
*zeol*	zeolyte

## References

[1] Smith A.R., Klosek J. (2001). A review of air separation technologies and their integration with energy conversion processes. Fuel Process. Technol..

[2] Luo L., Feidt M. (1992). Thermodynamics of adsorption cycles: A theoretical study. Heat Transf. Eng..

[3] Hashim S.S., Mohamed A.R., Bhatia E. (2011). Oxygen separation from air using ceramic-based membrane technology for sustainable fuel production and power generation. Renew. Sustain. Energy Rev..

[4] Hashim S.M., Mohamed A.R., Bhatia E. (2010). Current status of ceramic-based membranes for oxygen separation from air. Adv. Colloid Interface Sci..

[5] Lucia U. (2013). Adsorber efficiency in adsorption refrigeration. Renew. Sustain. Energy Rev..

[6] Jayaraman A., Yang R. (2005). Stable oxygen-selective sorbents for air separation. Chem. Eng. Sci..

[7] Talu O., Li J., Kumar R., Mathias P.M., Moyer J.D., Shork J.M. (1996). Measurement and analysis of oxygen/nitrogen/5A-zeolyte adsorption equilibria for air separation. Gas. Sep. Purif..

[8] Laredo G.C., Castillo J., Marroquin J. (2012). Dual-site Langmuir modeling of the liquid phase adsorption of linear and branched paraffins onto a PVDC carbon molecular sieve. Fuel.

[9] Talu O., Hayhurst D.T. (1989). Isosteric Heat of Adsorption by the Virial Isotherm Equation.

[10] Mathias P.M., Kumar R., Moyer J.D., Schork J.M., Srinivasan S.R., Auvil S.R., Talu O. (1996). Correlation of multicomponent gas adsorption by the dual-site langmuir model. Application to nitrogen/oxygen adsorption on 5a-zeolite. Ind. Eng. Chem. Res..

[11] Hemingway B.S., Robie R.A. (1984). Thermodynamic properties of zeolites: Low-temperature heat capacities and thermodynamic functions for phillipsite and clinoptilolite. Estimates of the thermochemical properties of zeolitic water at low temperature. Am. Mineral..

